# Toward the identification of cyano-astroCOMs via vibrational features: benzonitrile as a test case

**DOI:** 10.3389/fchem.2024.1439194

**Published:** 2024-09-03

**Authors:** Yanting Xu, Malgorzata Biczysko

**Affiliations:** International Centre for Quantum and Molecular Structures, Department of Physics, College of Sciences, Shanghai University, Shanghai, China

**Keywords:** vibrational spectra, density functional theory, second-order vibrational perturbation theory, near-infrared, James Webb Space Telescope

## Abstract

The James Webb Space Telescope (JWST) opened a new era for the identification of molecular systems in the interstellar medium (ISM) by vibrational features. One group of molecules of increasing interest is cyano-derivatives of aromatic organic molecules, which have already been identified in the ISM on the basis of the analysis of rotational signatures, and so, are plausible candidates for the detection by the JWST. Benzonitrile considered in this work represents a suitable example for the validation of a computational strategy, which can be further applied for different, larger, and not-yet observed molecules. For this purpose, anharmonic simulations of infrared (IR) spectra have been compared with recent FTIR experimental studies. The anharmonic computations using the generalized second-order vibrational perturbation theory (GVPT2) in conjunction with a hybrid force field combining the harmonic part of revDSD-PBEP86-D3/jun-cc-pVTZ with anharmonic corrections from B3LYP-D3/SNSD show very good agreement with those in the experiment, with a mean error of $11cm^{-1}$ for all fundamental transitions overall and only $2cm^{-1}$ for the C≡N stretching fundamental at 4.49 μm . The inclusion of overtones up to three-quanta transitions also allowed the prediction of spectra in the near-infrared region, which shows distinct features due to C≡N overtones at the 2.26 μm and 1.52 μm . The remarkable accuracy of the GVPT2 results opens a pathway for the reliable prediction of spectra for a broader range of cyano-astroCOMs.

## 1 Introduction

Despite being initially considered too harsh and diluted for molecule creation and survival, it is now recognized that several chemical processes can happen in astronomic environments leading to more than 310 molecules already detected in the interstellar medium (ISM) or circumstellar shells (Endres et al., 2016). Several of these molecules have been detected in the last decade, in particular, a number of complex organic species (COMs), defined as astrophysically relevant organic molecules consisting of six or more atoms. Some COMs that contain C, H, N, O, and possibly also S atoms can be claimed as prebiotic (Fulvio et al., 2021). To date, more than 30 prebiotic molecules have been detected in Taurus molecular cloud 1 (TMC-1), a dust-enshrouded gaseous cloud located 400 light-years from the Sun in the Taurus constellation. Such progress and fast increase in new detections has become possible due to advances in instrumentation, in particular the sub-millimeter and radio domains, allowing the analysis of the lowest rotational lines where radiation can pass through dust-enshrouded clouds (Guélin and Cernicharo, 2022).

**FIGURE 1:**
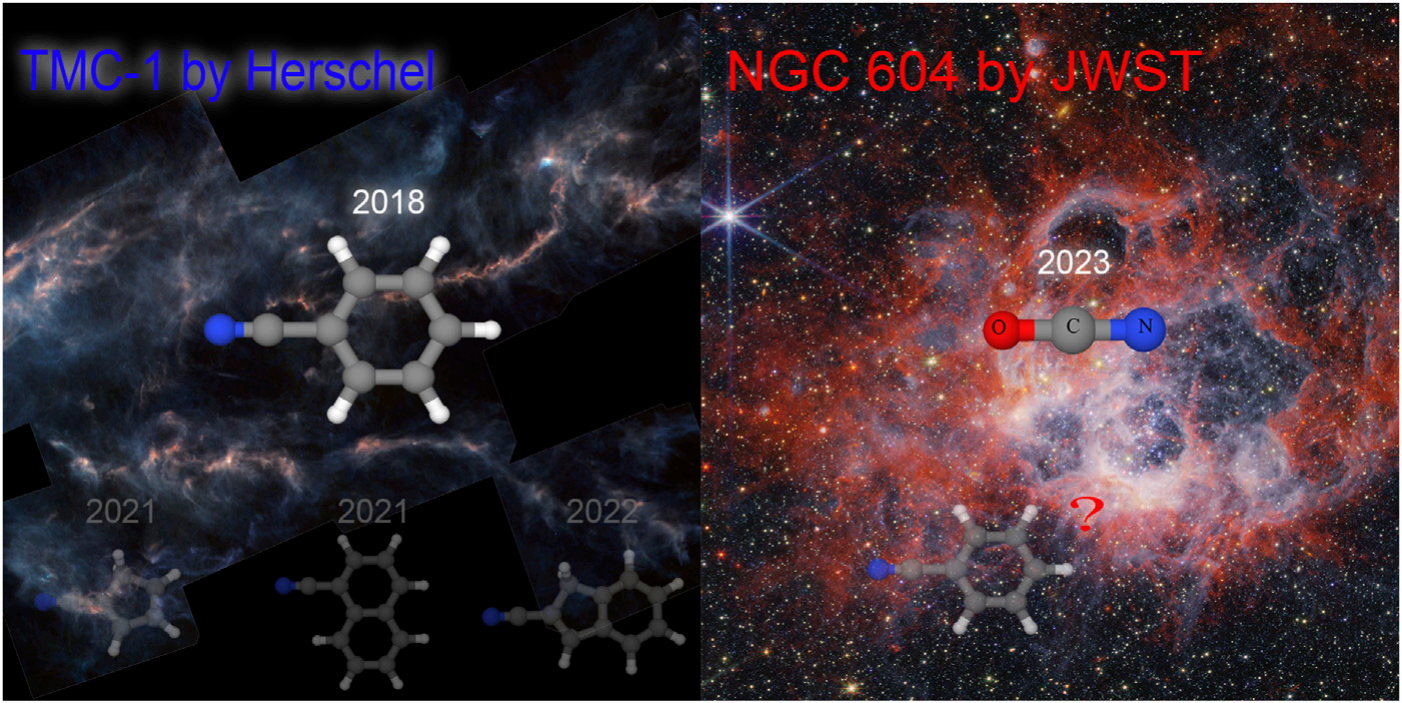
Cyano-derivatives in the ISM and the year of detection by rotational (left panel) or vibrational (right panel) features. Left panel: astro- COMs and Herschel’s view of TMC-1 (Herschel Space Observatory, 2017) (credit: ESA/Herschel/NASA/JPL-Caltech TMC-1). Right panel: ${OCN}^{-}$ recently detected by the JWST and NGC 604 NIRCam Image (Webb Space Telescope, 2024) (credit: NASA/ESA/CSA/STScI).

However, rotational transitions are not always suitable and accessible for a study, for instance, for exoplanet atmospheres or dense dark regions, for which vibrational spectroscopies are often the methods of choice. The increasing importance of vibrational transitions in astrochemical context is clearly represented by the James Webb Space Telescope (JWST), which, with the infrared (IR) (Frost et al., 2022) observations obtained by the mid-infrared (MIR) and near-IR (NIR) instruments integrated within the Integrated Science Instrument Module (ISIM, Near-Infrared Camera [NIRCam], Near-Infrared Spectrograph [NIRSpec], Mid-Infrared Instrument [MIRI], and Jet Propulsion Laboratory [JPL]), already provides superlative sensitivity, spectral resolution, and wavelength coverage compared to previous space telescopes, such as Herschel, that observe in the visible and ultraviolet spectra (see Figure 1). These emerging experimental data already provide new information about the major ices in molecular cloud cores just prior to their collapse to form protostars (McClure et al., 2023). Currently, the Mars 2020 Perseverance rover also searches for signs of organic matter, in the contexts of the emergence of life, as well as its consideration as a habitable planet, performing, among others, NIR studies using SuperCam (Perez et al., 2017; Eigenbrode et al., 2018; Lasne, 2021; Sharma et al., 2023; McIntosh et al., 2024). These missions yield a huge amount of new significant results, which need to be analyzed, also highlighting the urgent need for accurate reference data in the MIR-to-NIR (0.6–28 μm ) range (Öberg, 2016; Nazari et al., 2021; Zapata Trujillo et al., 2023; Fortenberry, 2024b).

Spectroscopic techniques are the key for the analysis of astronomical observations and the detection of molecules in the interstellar medium and other astrochemical environments, such as atmospheres or soils of exoplanets or planetary moons (Loru et al., 2022; Lemmens et al., 2022; Mackie et al., 2022; Peeters et al., 2021; K. Lemmens et al., 2023; Petrignani and Candian, 2022; Puzzarini et al., 2023). However, the investigation of the chemical composition of “astrochemical samples” is complicated due to the concomitant presence of many, possibly unknown species. The detection of molecules is based on the comparison of spectra from spectroscopic observations in space with reference experiments from the laboratory (Puzzarini et al., 2023). Unfortunately, the latter might be limited, incomplete, or difficult to obtain under appropriate conditions (Barone et al., 2015c; Fortenberry, 2024b). The optimal strategy is represented by the combination of experiments with the theoretical approaches (Barone et al., 2021; Puzzarini, 2022), supporting and/or complementing laboratory studies (Biczysko et al., 2018a; Barone and Puzzarini, 2023; Fortenberry, 2024a). An increasing role of computational spectroscopy is related to its increasing accuracy (Yang et al., 2021; Barone and Puzzarini, 2023), as well as the possibility to match different, often extreme environmental conditions possible in space (Barone et al., 2015c; Biczysko et al., 2018b; Zapata Trujillo et al., 2023; Fortenberry, 2024b).

Among all the molecules detected to date in the ISM, over 30% bear a nitrogen atom, which usually bonds to carbon, in a large fraction (over 80% of species) by the triple C ≡ N bond (Endres et al., 2016). This is in line with the increased dipole moment that facilitates detection by rotational features, as well as the abundance of the CN radical in the ISM (McGuire, 2022) and its extreme reactivity (McGuire et al., 2018; 2021) leading to the abundance of cyano-substituted derivatives (cyano-astroCOMs and C ≡ N-astroCOMs) in the ISM. Indeed, benzonitrile (B C≡N , cyano-benzene, $C_{6}$ H_5_C ≡ N), was the first six-membered aromatic compound detected in 2018 in the ISM (McGuire et al., 2018), toward the dark molecular cloud TMC-1, a well-studied region where most molecules were first observed. This detection was possible as the C ≡ N group attachment creates a permanent dipole moment, which is null in otherwise “silent” (poly-)aromatic hydrocarbons (PAHs). This breakthrough intensified the search for aromatic C ≡ N-astroCOMs, leading to the detection in the TMC-1 of 1- and 2-cyanonaphthalene ( $C_{10}$ H_7_C ≡ N) isomers (McGuire et al., 2021) in 2021 and 2-cyanoindene ( $C_{9}$ H_7_C ≡ N) (Sita et al., 2022) in 2022. Following the observation of the six-membered rings, 1- and 2-cyanocyclopentadiene ( $C_{5}$ H_5_C ≡ N) (McCarthy et al., 2021; Lee et al., 2021), which contains a five-membered cycle, were also detected in the same source (see Figure 1). Further searches extending to other hetero-aromatic and bridged bicyclic compounds (Martin-Drumel et al., 2023) also aim at establishing the abundance ratio between C ≡ N-PAHs and their parent PAHs (Barone and Lazzari, 2023). To date, none of these molecules have been identified by their vibrational features, but the first cyano-compound, i.e., the O C≡N ^-^ anion, which is one of the most well-detected species in astrophysical ices, has been detected in the low-mass star-forming region ChameleonI by the JWST based on the peak centered at $2165.9cm^{-1}$ (4.62 μm ) (McClure et al., 2023) (see Figure 1).

In this work, we focus on the first observed cyano-astroCOM, i.e., benzonitrile (McGuire et al., 2018). The experimental microwave spectrum of benzonitrile was first studied simultaneously by Erlandsson (1954) and Lide (1954). Bak et al. conducted comprehensive analyses of benzonitrile utilizing infrared and centimeter-wave techniques, also including feasible mono-substituted isotopologs (Bak and Nielsen, 1960; Bak et al., 1962). This led to the first derivation of a substitution structure, $r_{s}$ , which was further refined by Casado et al. by incorporating multiple Q-branch transitions for the majority of mono-substituted isotopologs (Casado et al., 1971). Green and Harrison subsequently enhanced the analysis of the experimental IR spectrum, taking into account the lowest wavenumber modes (Green and Harrison, 1976). Recently, these initial spectroscopic investigations have been improved, also in view of the increasing accuracy and spectrum coverage requirements due to the astrochemical importance of B C≡N (Yamamoto et al., 2000; Kwon et al., 2003; Burova and Anashkin, 2007; Rajasekhar et al., 2022). The accurate equilibrium structure of benzonitrile has been determined by Rudolph et al. (2013) using two different, complementary techniques, namely, the theoretical estimate $r_{BO}$ and semi-experimental $r_{e}^{SE}$ . The most extended study is the 2022 synchrotron investigation of B C≡N in a very broad spectrum range, up to $90^{\prime }000cm^{-1}$ (11.1 eV), which also included the new detection of the gas-phase IR spectra at a resolution of $0.5cm^{-1}$ (Rajasekhar et al., 2022). This work, along with the high-resolution far-infrared spectra collected in the $65--695cm^{-1}$ range using synchrotron radiation at the SOLEIL facility (Zdanovskaia et al., 2022), represents the reference for the anharmonic computations of IR spectra.

This work focuses on the IR spectra in the MIR-NIR region, first comparing the simulated infrared spectra with the available experimental counterparts and then providing the prediction of not yet available spectral data, which can support either laboratory or astrochemical studies.

## 2 Computational details

In order to simulate the spectroscopic parameters of benzonitrile in its electronic ground state ${\tilde{X}}^{1}A_{1}$ , the geometry optimization and harmonic and anharmonic vibrational computations are performed using GAUSSIAN 16 (Frisch et al., 2016). In geometry optimization, the tight convergence criteria (maximum forces and displacements smaller than $1.5\times 10^{-5}$ Hartree/Bohr and $6.0\times 10^{-5}$ Å, respectively), as required for the anharmonic computations, are used. The equilibrium structure, harmonic force constants, and first-order electric dipole moment derivatives have been computed using the double-hybrid density functional revDSD-PBEP86 (Santra et al., 2019), which has been recommended for spectroscopic studies of medium-sized biomolecules (Barone et al., 2020; Yang et al., 2021; Mehta et al., 2023). These computations have been performed in conjunction with the jun-cc-pVTZ (denoted hereafter as junTZ) basis set (Papajak et al., 2011), which provides the optimal accuracy/cost ratio, as recently discussed by Xu et al. (2024). Moreover, the B3LYP (Becke, 1993)/SNSD (Barone et al., 2014) level has been used in the anharmonic computations. For both density functional theory (DFT) functionals, the dispersion correction proposed by Grimme (2011) has been added using the D3 (Grimme et al., 2010) version with Becke–Johnson (BJ) damping (Grimme et al., 2011; Najibi and Goerigk, 2018). For brevity, hereafter, the revDSD-PBE86-D3/jun-cc-pVTZ and B3LYP-D3/SNSD levels will be denoted as revDSD and B3LYP. 

Computations of the third- and fourth-order derivatives of the potential energy surface have been performed at the B3LYP level by the numerical differentiation (Barone, 2005; Bloino, 2015) of analytic second-order derivatives, while the cubic electric dipole moment surfaces have been obtained through numerical differentiations of the dipole moment derivatives. The revDSD equilibrium and harmonic computations have been combined with B3LYP anharmonic computations to create a hybrid model used in spectroscopic simulations. The consistency of these two sets of data has been checked automatically, as implemented in GAUSSIAN 16. In other words, the overlap between two sets of normal modes (two different levels of theory) is defined using the linear transformation, as proposed by Duschinsky (1937):

$$Q=JQ^{\prime }+K,$$

where Q and $Q^{\prime }$ represent the two sets of mass-weighted normal coordinates. The Duschinsky matrix J describes the projection of normal coordinate basis vectors on those of the other, allowing the automatic check of the normal mode consistency between the two levels of theory used to define the hybrid method. To ensure that the two sets of normal modes computed at different levels of theory are equivalent, a 90% cut-off for each coordinate was required.

This cost-effective (Xu et al., 2024) hybrid revDSD/B3LYP scheme has been further used to compute spectroscopic parameters using the second-order vibrational perturbation theory (VPT2) (Nielsen, 1951; Mills, 1972). The ground vibrational state rotational constants have been obtained from the revDSD equilibrium structure by adding vibrational corrections computed at the revDSD/B3LYP level, which also provided data allowing the determination of the quartic and sextic centrifugal-distortion constants (Puzzarini et al., 2010; Puzzarini, 2013). For the vibrational spectra, it is also necessary to account for the possible presence of anharmonic resonances (Amos et al., 1991; Martin et al., 1995; Barone, 2005; Vázquez and Stanton, 2007; Rosnik and Polik, 2014; Barone et al., 2014; Bloino et al., 2015; Bloino, 2015; Krasnoshchekov et al., 2015; Franke et al., 2021; Mendolicchio et al., 2021; Franke et al., 2021) by the generalized VPT2 (GVPT2) model (Bloino and Barone, 2012; Bloino et al., 2015), where nearly resonant contributions are removed from the perturbative treatment (leading to the deperturbed model, DVPT2) and treated in a second step variationally. Resonance definition and general recommendations on the applied computational procedures are described in detail in the tutorial review by Bloino et al. (2016). It should be noted that, although improved criteria to define automatic resonances have been proposed recently, they would have negligible impacts on the energies (Yang and Bloino, 2022). Overall, the GVPT2 scheme employed in this work has been successfully applied to medium-sized or larger biomolecules with up to 100 atoms (Fornaro et al., 2015; Fusè et al., 2019; Yatsyna et al., 2019; Green and Improta, 2020; Yang et al., 2021; Sheng et al., 2021), also in the astrochemical context (Zhao et al., 2021; McIntosh et al., 2024; Alberini et al., 2024), so it is a valuable tool to be employed also for significantly larger cyano-astroCOMs.

## 3 Results and discussion

### 3.1 Equilibrium structure and rotational parameters

**FIGURE 2:**
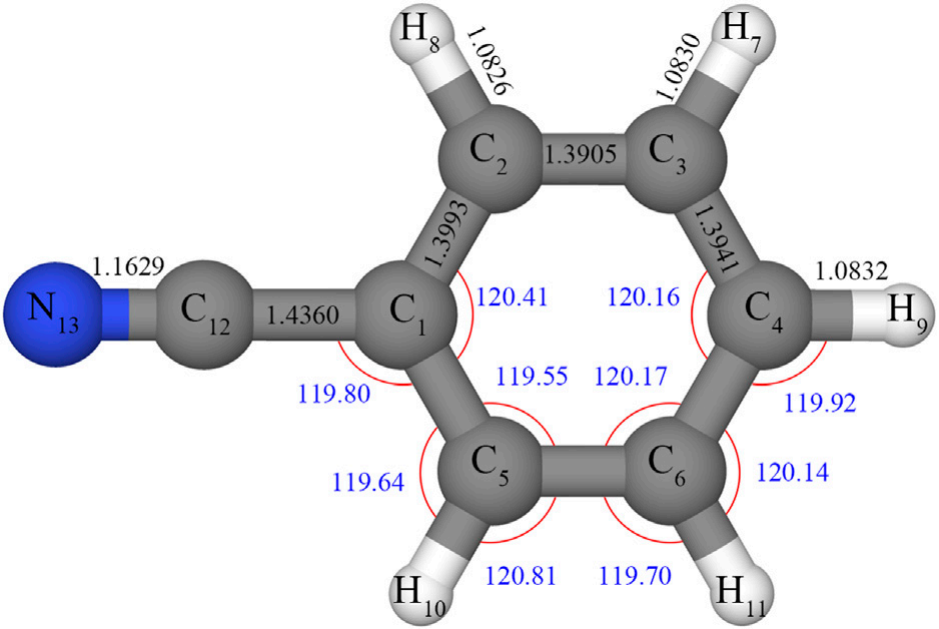
Structural parameter of benzonitrile as computed at the rDSD/junTZ level, with bond lengths (black) in Å and angles (blue) in degrees.

Selected equilibrium structural parameters of benzonitrile calculated at the revDSD/junTZ level are shown in Figure 2, while Table 1 compares all equilibrium parameters with the semi-experimental equilibrium structure $r_{e}^{SE}$ (Pulay et al., 1978) derived in the reference (Rudolph et al., 2013) by combining the experimental ground-state rotational constants for a set of isotopologs with rovibrational corrections derived from cubic force fields determined at the B3LYP level (Piccardo et al., 2015). Moreover, Table 1 reports the best estimated theoretical structure $r_{BO}$ obtained using the composite scheme employing all-electron CCSD(T) and MP2 geometry optimizations, with basis sets up to the quintuple-zeta, reaching this way the complete basis set (CBS) limit, as well as computations at the CCSD(T)/ANO1 level (Rajasekhar et al., 2022). Structural parameters computed by all combinations of revDSD and B3LYP functionals with the junTZ and SNSD basis sets are provided in Supplementary Material, along with the Cartesian coordinates by revDSD/junTZ. All DFT structures agree very well with the $r_{e}^{SE}$ reference, among which revDSD/junTZ shows the smallest mean absolute errors (MAEs) of approximately 0.0030 Å for bond lengths and 0.08° for the angles, which, in terms of the largest discrepancies, correspond to approximately 0.005 Å and ± 0.15°, respectively. The good quality structure with MAEs of 0.0045 Å and 0.13°, respectively, is also observed for B3LYP/SNSD, justifying its application in the hybrid scheme. Interestingly, the revDSD/junTZ structure is closer to the $r_{e}^{SE}$ reference than the CCSD(T)/ANO1 structure, further proving the reliability of revDSD as a cost-effective computational model, allowing to derive accurate geometrical parameters (Ceselin et al., 2021; Barone and Lazzari, 2023).

**TABLE 1:** Equilibrium parameters of benzonitrile (bond lengths in Å; angles in degrees).

	$r_{e}^{SE}$ ^a^	$r_{e}^{BO}$ ^b^	CCSD(T)/ANO1^c^	rDSD/junTZ
$C_{1}$ - $C_{2}$	1.3968	1.3962	1.4012	1.3993
$C_{2}$ - $C_{3}$	1.3884	1.3882	1.3934	1.3905
$C_{3}$ - $C_{4}$	1.3917	1.3917	1.3967	1.3941
$C_{1}$ - $C_{12}$	1.4347	1.4359	1.4393	1.4360
$C_{2}$ -H_8_	1.0780	1.0803	1.0823	1.0826
$C_{3}$ -H_7_	1.0799	1.0803	1.0824	1.0830
$C_{4}$ -H_9_	1.0800	1.0806	1.0828	1.0832
$C_{12}$ -N_13_	1.1582	1.1583	1.1646	1.1629
| MAX ${|}^{d}$	.	0.0023	0.0064	0.0047
MAE^d^	.	0.0007	0.0043	0.0030
$C_{1}$ C_2_C_3_	119.42	119.52	119.54	119.55
$C_{2}$ C_3_C_4_	120.27	120.13	120.13	120.17
$C_{3}$ C_4_C_6_	120.07	120.22	120.19	120.16
$C_{2}$ C_1_C_5_	120.55	120.50	120.46	120.41
$C_{1}$ C_2_H_8_	119.77	119.61	119.61	119.64
$C_{4}$ C_3_H_7_	120.10	120.16	120.16	120.14
| MAX ${|}^{d}$	.	0.16	0.16	0.15
MAE^d^	.	0.09	0.09	0.08

^a^Semi-experimental $r_{e}^{SE}$ from Rudolph et al. (2013), obtained by combining the experimental ground-state rotational constants for a set of isotopologs with rovibrational corrections derived from cubic force fields determined at the B3LYP level.

^b^ Theoretical best estimated from $r_{e}^{BO}$ Rudolph et al. (2013), obtained using a composite *ab initio* approach based on CCSD(T) and MP2 all-electron optimizations with basis sets up to quintuple-zeta quality.

^c^ Theoretical structure obtained at the CCSD(T)/ANO1 level from Zdanovskaia et al. (2022).

^d^ Largest absolute ( | MAX | ) and mean absolute errors (MAEs) of the bond length and angles compared to the $r_{e}^{SE}$ from Rudolph et al. (2013).

The final validation is provided by the direct comparison with the experiment, i.e., spectroscopic constants from Watson’s asymmetric rotor Hamiltonian (A-reduction, $I^{r}$ representation), which are given in Table 2. Interestingly, the vibrational ground-state rotational constants obtained based on the revDSD structures with anharmonic corrections computed at the revDSD/B3LYP level agree with experiment within 0.4%, which is even better than the CCSD(T)/ANO1 obtained by Zdanovskaia et al. (2022). Moreover, good agreement, again similar to the CCSD(T)/ANO1 results, is also obtained for the quartic and sextic centrifugal-distortion constants. Notably, in some cases, such as the $\Phi_{J}$ sextic constant, very good agreement with the most recent global fit including previous and expanded mm-wave measurements (Zdanovskaia et al., 2022) is obtained, while older experimental data reported values smaller by 50%.

**TABLE 2:** Rotational spectroscopic constants for the ground vibrational state of benzonitrile.

	Experiment^a^	CCSD(T)/ANO1^b^	rDSD/junTZ//B3LYP/SNSD
$A_{0}$ (MHz)	5,655.265,428	5,616.	5,638.
$B_{0}$ (MHz)	1,546.8757715	1535.	1,541.
$C_{0}$ (MHz)	1,214.4040832	1,205.	1,210.
$\Delta_{J}$ (kHz)	0.0452858	0.0437	0.0433
$\Delta_{JK}$ (kHz)	0.937983	0.923	0.922
$\Delta_{K}$ (kHz)	0.24411	0.241	0.226
$\delta_{J}$ (kHz)	0.01101116	0.0106	0.0106
$\delta_{K}$ (kHz)	0.609187	0.593	0.592
$\Phi_{J}$ (Hz)	0.000002486	0.00000230	0.00000226
$\Phi_{JK}$ (Hz)	0.0015586	0.00149	0.00150
$\Phi_{KJ}$ (Hz)	-0.007863	-0.00761	-0.00769
$\Phi_{K}$ (Hz)	[0.0066915]	0.0066915	0.0067529
$\phi_{J}$ (Hz)	0.000001159	0.00000110	0.00000105
$\phi_{JK}$ (Hz)	0.0007398	0.000755	0.000757
$\phi_{K}$ (Hz)	0.007480	0.00712	0.00714

a Spectroscopic constants derived by Zdanovskaia et al. (2022) using the single-state approach based on the ground state

^b^Spectroscopic constants obtained by Zdanovskaia et al. (2022) using the VPT2 computations at the CCSD(T)/ANO1 level.

### 3.2 Vibrational properties and IR spectra

Table 3 compares harmonic vibrational wavenumbers and IR intensities with those computed at the CCSD(T)/ANO1 level, showing very good agreement with the average error of approximately 6 $cm^{-1}$ and largest differences of approximately $18cm^{-1}$ , as well as qualitative agreement for IR intensities, with a MAE below 1 km/mol, and largest discrepancies of approximately ± 7 km/mol observed for the most intense bands $\nu_{18}$ and $\nu_{19}$ . Thus, the good accuracy of revDSD harmonic wavenumbers is also demonstrated for benzonitrile, in accordance with what has been observed based on the comparison with CCSD(T) results with the CBS extrapolation from MP2 computations (Pietropolli Charmet et al., 2022; Tasinato et al., 2022; Xu et al., 2024). In Table 3 and following, we have adapted mode numbering, mode description, and Wilson notation, as done by Rajasekhar et al. (2022).

**TABLE 3:** Harmonic wavenumbers $\left(\omega ,cm^{-1}\right)$ and IR intensities (km/mol) compared with reference computed data.

Sym	CCSD(T)/ANO1^a^		rDSD/junTZ	
	ω	IR int.	ω	IR int.
A1				
$\nu_{1}$	3,219	3.53	3,221	3.85
$\nu_{2}$	3,206	5.43	3,208	6.29
$\nu_{3}$	3,189	0.01	3,189	0.00
$\nu_{4}$	2,277	6.14	2,263	11.32
$\nu_{5}$	1,645	0.12	1,653	0.12
$\nu_{6}$	1,520	9.82	1,530	8.75
$\nu_{7}$	1,213	0.16	1,221	0.30
$\nu_{8}$	1,195	0.55	1,202	0.72
$\nu_{9}$	1,043	2.67	1,051	3.34
$\nu_{10}$	1,010	0.12	1,018	0.23
$\nu_{11}$	763	1.38	769	1.49
$\nu_{12}$	459	0.00	462	0.00
A2				
$\nu_{13}$	987	0.00	996	0.00
$\nu_{14}$	860	0.00	866	0.00
$\nu_{15}$	402	0.00	405	0.00
B1				
$\nu_{16}$	1,006	0.01	1,009	0.00
$\nu_{17}$	939	2.81	945	2.83
$\nu_{18}$	769	48.33	770	56.01
$\nu_{19}$	696	26.77	679	19.74
$\nu_{20}$	550	15.42	556	15.05
$\nu_{21}$	378	0.69	382	0.64
$\nu_{22}$	143	1.70	144	1.79
B2				
$\nu_{23}$	3,214	6.27	3,217	6.86
$\nu_{24}$	3,198	1.94	3,200	2.52
$\nu_{25}$	1,620	1.33	1,628	1.24
$\nu_{26}$	1,471	6.71	1,481	6.18
$\nu_{27}$	1,348	0.96	1,358	1.15
$\nu_{28}$	1,314	1.97	1,329	1.46
$\nu_{29}$	1,175	0.26	1,182	0.24
$\nu_{30}$	1,096	2.96	1,104	3.60
$\nu_{31}$	629	0.12	633	0.12
$\nu_{32}$	546	0.14	552	0.24
$\nu_{33}$	162	4.44	162	4.65
MAX	-		15	7.7
MIN	-		-18	-7.0
MAE	-		6	0.8

^a^ Ref. (Zdanovskaia et al., 2022).

^b^ Largest positive (MAX), negative (MIN), and mean absolute errors (MAEs) of the harmonic wavenumbers compared with the CCSD(T)/ANO1 reference (Zdanovskaia et al., 2022).

**FIGURE 3:**
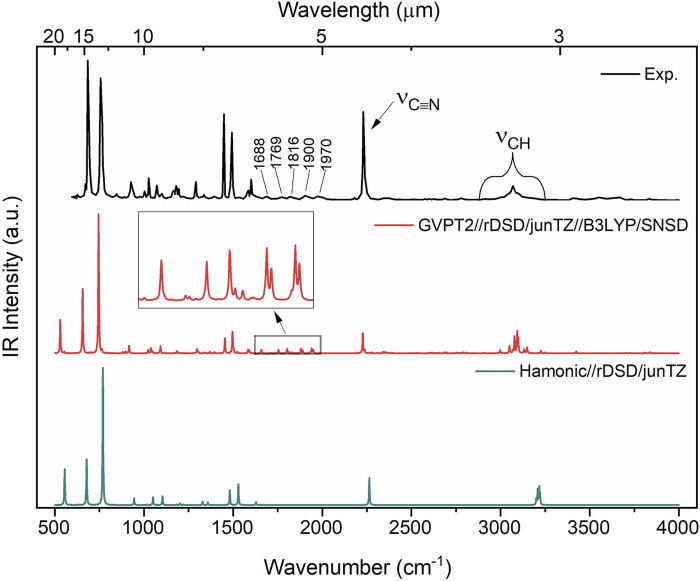
IR spectrum of benzonitrile in the range 500–4,000 $cm^{-1}$ . Computed stick spectra were broadened by Lorentzian functions with half-width at half-maximum (HWHM) of 2 $cm^{-1}$ . The experimental IR gas-phase spectrum of benzonitrile (Rajasekhar et al., 2022) is shown for comparison.

Table 4 lists all fundamental anharmonic wavenumbers and IR intensities of benzonitrile computed at the rDSD/junTZ//B3PLYP/SNSD GVPT2 level, while selected overtones and combination bands are given in Table 5. The accuracy of the simulated IR spectra of benzonitrile in the 500 $cm^{-1}$ –4,000 $cm^{-1}$ range can be assessed by comparing with experimental results recorded in the gas phase (Kwon et al., 2003; Rajasekhar et al., 2022). Table 4 provides both spectra, with the latter showing a higher resolution of $0.5cm^{-1}$ . This increased resolution allows us to identify and assign non-fundamental transitions, as shown in Figure 3. The GVPT2 computations show overall good agreement with the experiment with a MAE of approximately $11cm^{-1}$ and the largest positive and negative errors of approximately ± $44cm^{-1}$ . Moreover, the largest errors are all related to the C-H stretching vibrations, which contribute to the broad band with some additional side peaks, which has not been assigned. Considering that our simulation agrees within $12cm^{-1}$ with the most intense peak assigned as $\nu_{2}$ , it could be expected that further analysis of experimental data, including non-fundamental transitions, would lead to some re-assignments. Excluding all C-H stretching vibrations from the statistics leads to the average errors of $8cm^{-1}$ and maximum discrepancies within $31cm^{-1}$ . The most important result is extremely good agreement, within $2cm^{-1}$ , for the $\nu_{C\equiv N}$ , the fingerprint vibration of benzonitrile, which is predicted at $2227cm^{-1}$ (4.49 μm ). This result can be compared with the very recent study where the B3LYP/N07D quadratic force field was combined with VPT2 computations with resonances included (see Esposito et al. (2024) for details) but performed with the SPECTRO code, yielding $\nu_{C\equiv N}$ of $2298cm^{-1}$ . Indeed, GVPT2 B3LYP-D3/N07D computations in GAUSSIAN 16 lead to a similar result, with $\nu_{C\equiv N}$ of $2305cm^{-1}$ . Such a huge discrepancy of approximately $70cm^{-1}$ was not expected based on previous benchmark tests, highlighting the need for a dedicated validation. We hope that the proposed GVPT2 revDSD/B3LYP methodology will allow us to distinguish between the different cyano-astroCOMs observed in the ISM using the $\nu_{C\equiv N}$ vibrations normally occurring in the broader region 2,200–2,400 $cm^{-1}$ (Császár and Fogarasi, 1989).

**TABLE 4:** Fundamental wavenumbers $\left(\upsilon ,cm^{-1}\right)$ and IR intensities (km/mol) computed at the GVPT2//revDSD/junTZ//B3LYP/SNSD level for benzonitrile compared with reference experimental and computed data.

Sym	Mode description^a^ Wilson notation		Ref.^a^		Ref.^b^			Current work		Assignments (PED)^c^
			Expt	Theory^d^	Expt	Theory^e^	IR Int. scaled	υ	IR int	
A1										
$\nu_{1}$	20a	ν CH	3,080	3,207	3,106	3,210	2.40	3,148	2.19	ν CH
$\nu_{2}$	2	ν CH	3,071	3,196	3,066	3,208	6.54	3,078	3.90	ν CH
$\nu_{3}$	7a	ν CH	3,042	3,178	3,043	3,198	8.46	2,999	1.20	ν CH
$\nu_{4}$	ν CN	ν CN	2,232	2,332	2,229	2,323	34.02	2,227	7.44	ν CN, ν CC
$\nu_{5}$	8a	ν CC	1,599	1,641	1,599	1,643	0.62	1,610	0.01	ν CC, β CCH
$\nu_{6}$	19a	ν CC	1,492	1,582	1,491	1,509	8.39	1,496	6.98	β HCC, β R sym, ν CC
$\nu_{7}$	13	X-sens	1,191	1,220	1,193	1,214	0.33	1,202	0.08	ν CC, β CCH, β R sym
$\nu_{8}$	9a	Ring	1,178	1,203	1,178	1,192	0.93	1,185	0.84	β CCH, ν CC
$\nu_{9}$	18a	β CH	1,027	1,050	1,027	1,045	3.79	1,039	1.90	ν CC, β R sym, β CCH
$\nu_{10}$	12	β CH	1,001	1,019	1,001	1,010	0.00	1,008	0.14	ω CCCH, τ R asy
$\nu_{11}$	1	X-sens	769	774	758	769	2.05	764	1.11	β R tri, ν CC
$\nu_{12}$	6a	X-sens	461	467	459	463	0.00	458	0.00	β R tri, ν CC
A2										
$\nu_{13}$	17a	γ CH	978	1,002	975	984	0.00	968	0.00	ω CCCH, τ R asy
$\nu_{14}$	10a	γ CH	848	863	844	849	0.00	841	0.00	ω CCCH
$\nu_{15}$	16a	ϕ CC	401	410	398	408	0.00	395	0.00	τ R asy, ω CCCH
B1										
$\nu_{16}$	5	γ CH	987	1,021	1,001	1,008	0.37	977	0.00	β R asy, ν CC
$\nu_{17}$	17b	γ CH	925	954	926	935	2.50	917	2.86	ω CCCH
$\nu_{18}$	11	γ CH	758	781	758	773	34.79	746	50.72	ω CCCH, τ R tri, ω CCCC
$\nu_{19}$	4	ϕ CC	686	706	687	704	38.87	656	23.67	τ R asy, ω CCCH
$\nu_{20}$	16b	X-sens	548	573	547	566	17.79	531	12.09	β CCN, ω CCCH, τ R asy
$\nu_{21}$	γ CN	X-sens	381	392	372	388	0.61	371	0.72	τ R asy, β CCN
$\nu_{22}$	10b	γ CN	172	147	141	147	1.93	138	1.90	ω CCCC, β CCN, τ R tri
B2										
$\nu_{23}$	20b	ν CH	3,039	3,188	3,093	3,208	6.54	3,093	7.49	ν CH
$\nu_{24}$	7b	ν CH	3,027	3,204	3,027	3,190	3.95	3,068	0.86	ν CH
$\nu_{25}$	8b	ν CC	1,584	1,615	1,583	1,614	0.80	1,591	0.89	ν CC
$\nu_{26}$	19b	ν CC	1,448	1,481	1,448	1,462	6.69	1,454	5.73	β CCH, ν CC
$\nu_{27}$	14	ν CC	1,337	1,361	1,335	1,351	1.79	1,337	0.25	ν CC, β CCH
$\nu_{28}$	3	β CH	1,298	1,319	1,288	1,322	0.46	1,298	1.58	β CCH, ν CC
$\nu_{29}$	9b	β CH	1,163	1,188	1,163	1,176	0.16	1,172	0.13	β CCH, ν CC
$\nu_{30}$	18b	β CH	1,071	1,105	1,071	1,099	4.35	1,093	2.52	β CCH, ν CC
$\nu_{31}$	6b	α CCC	629	641		633	0.12	628	0.11	β R sym
$\nu_{32}$	β CN	β CN	551	570	547	559	0.27	547	0.09	β CCC, ω CCCN
$\nu_{33}$	15	X-sens	162	169		167	4.65	158	4.69	β R asy, ω CCCN
All		MAX^f^	42							
		MIN^f^	-44							
		MAE^f^	11							
Exclude all ν CH		MAX^g^	22							
		MIN^g^	-31							
		MAE^g^	8							

^a^ Ref (Kwon et al., 2003).

^b^ Ref (Rajasekhar et al., 2022).

^c^ Normal mode assignments, ν , β , ω , τ , and tri denote the stretching, in-plane bending, out-of-plane bending, torsion, and trigonal deformation, respectively. “sym” and “asy” stand for symmetrical and asymmetric deformation, respectively.

^d^ Scaled harmonic computations at the B3LYP/6-311++G(2df,2pd) level of theory.

^e^ Scaled harmonic computations at the B3LYP/aug-cc-pVDZ level of theory.

^f^ Largest positive (MAX), negative (MIN), and mean absolute errors (MAEs) of the benzonitrile fundamental wavenumbers compared with the experiment by Rajasekhar et al. (2022).

^g^ Largest positive (MAX), negative (MIN), and mean absolute errors (MAEs) in the benzonitrile fundamental wavenumbers, with all C-H stretching excluded, compared with the experiment by Rajasekhar et al. (2022).

**TABLE 5:** Non-fundamental band wavenumbers $\left(\upsilon ,cm^{-1}\right)$ and IR intensities (km/mol) computed at the GVPT2//revDSD/junTZ//B3LYP/SNSD level for benzonitrile compared with reference experimental data.

Experiment		Current work		
Assign.	υ	Assign.	υ	IR int.
2 $\nu_{22}$	282^a^	2 $\nu_{22}$	275	0.007
$\nu_{22}$ + $\nu_{33}$	303^a^	$\nu_{22}$ + $\nu_{33}$	296	0
2 $\nu_{33}$	323^a^	2 $\nu_{33}$	315	0.01
2 $\nu_{32}$ +2 $\nu_{22}$	1,393^b^	$\nu_{18}$ + $\nu_{19}$	1,397	0.41
2 $\nu_{18}$ + $\nu_{22}$	1,688^b^	$\nu_{17}$ + $\nu_{18}$	1,658	1.37
$\nu_{26}$ + $\nu_{22}$	1,769^b^	$\nu_{14}$ + $\nu_{17}$	1,754	1.30
$\nu_{26}$ +3 $\nu_{22}$	1,816^b^	$\nu_{13}$ + $\nu_{14}$	1,803	1.69
$\nu_{26}$ + $\nu_{12}$	1,900^b^	$\nu_{16}$ + $\nu_{17}$ , $\nu_{13}$ + $\nu_{17}$	1,883	2.83
$\nu_{26}$ + $\nu_{32}$	1,970^b^	$\nu_{13}$ + $\nu_{16},2\nu_{15}$	1,942	3.23
$\nu_{32}$ + $\nu_{18}$	2,178^b^	2 $\nu_{30}$	2,178	0.02

a High-resolution IR spectra in the gas phase obtained by Zdanovskaia et al. (2022).

b Gas-phase IR spectra obtained by Rajasekhar et al. (2022).

A direct comparison between the spectra given in Figure 3 highlights that the GVPT2 computation not only correctly predicts fundamental bands but also a pattern of five distinct non-fundamental bands in the $1650-1980cm^{-1}$ range, allowing to correct their assignment with respect to the tentative one reported by Rajasekhar et al. (2022). These non-fundamental bands are reported in Table 5, together with those observed in the far-infrared spectra in a high-resolution FTIR experiment (Zdanovskaia et al., 2022). The overestimated intensity of the $\nu_{18}$ band at the $758cm^{-1}$ should be noted (computed as $746cm^{-1}$ ), while the experimental spectra show two similar intensity peaks in this range, the second one being $\nu_{19}$ at $686cm^{-1}$ . However, this discrepancy needs to be linked to the harmonic values, which already predict the intensity of $\nu_{18}$ as twice that of $\nu_{19}$ . Notably, CCSD(T)/ANO-1 harmonic IR intensities yield the same pattern of these two bands as revDSD/junT. In order to provide more information about this discrepancy, a dedicated benchmark analysis, which would require appropriate numerical data on integrated intensities Charmet et al. (2013), not available at present, would be required.

**FIGURE 4:**
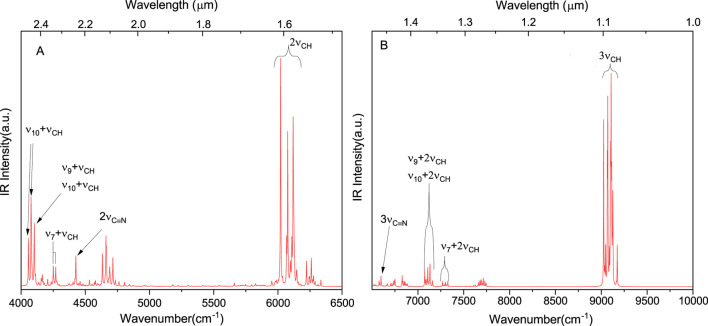
NIR spectra simulated at the GVPT2//rDSD/junTZ//B3LYP/SNSD level. The spectra were broadened by Lorentzian functions with HWHM of 2 $cm^{-1}$ .

Overall, the good accuracy of our simulations, for both fundamental and non-fundamental transitions, allows us to predict the spectra in the NIR region, which is shown in Figures 4A and B for $4000-6500cm^{-1}$ and $6500-10000cm^{-1}$ , respectively (the whole $100-9000cm^{-1}$ spectrum is also reported in Supplementary Material). The most pronounced bands in $4040-4300cm^{-1}$ and $6000-6180cm^{-1}$ are related to the combinations of $\nu_{CH}$ with in-plane ring deformations and $2\nu_{CH}$ overtones, respectively. Similarly, at the higher energies, there are combinations of $\nu_{CH}$ two quanta transitions with in-plane ring deformations ( $7070-7350cm^{-1}$ ) and 3 $\nu_{CH}$ second overtones ( $9000-9200cm^{-1}$ ). Although it is expected that the accuracy of GVPT2 results decreases for the higher-quanta transitions, the error bars for the first and second overtones can be estimated based on the fundamental bands (Barone et al., 2015a). For instance, in the case of formaldehyde (Biczysko et al., 2018a), good accuracy within $1cm^{-1}$ has been obtained for fundamentals and first overtones of $\nu_{6}$ and $\nu_{2}$ , while a lower accuracy of $\nu_{4}$ of approximately $10cm^{-1}$ transfers to errors of $20cm^{-1}$ and $26cm^{-1}$ for 2 $\nu_{4}$ and 3 $\nu_{4}$ , respectively. This allows us to provide a reliable prediction of $\nu_{C\equiv N}$ overtones, with distinct 2 $\nu_{C\equiv N}$ transition at 4,426 $\pm 2cm^{-1}$ (2.26 μm ) and a significantly weaker second overtone 3 $\nu_{C\equiv N}$ at 6,598 $\pm 5cm^{-1}$ (1.52 μm ).

## 4 Conclusion and astrochemical implications

Good accuracy of simulated MIR spectra, confirmed by a comparison with available experimental results, allowed us to provide predictions regarding the “missing” data on relevance for the astrochemical observations, in particular, concerning the NIR region.

The availability of NIR references is important for the interpretation of data collected during the Mars 2020 (Williford et al., 2018) space mission by instruments such as SuperCam, for incoming ExoMars 2022 (ESZ-Roscosmos) (Vago et al., 2017), as well as for the JWST observations by the NIRSpec (McClure et al., 2023). The advantage of NIR is its lower spectrum congestion than MIR, which is also clearly visible in Figures 3, 4. However, these reference NIR data are often very scarce and were not available even for benzonitrile prior to this work. It has been already highlighted that anharmonic computations provide significant support in the analysis of experimental results and identification of plausible molecules by NIR features (Fornaro et al., 2020; Alberini et al., 2024).

Computational spectroscopy can also support the identification of B C≡N in other spectral regions, also considering effects due to the interactions with cosmic rays (Öberg, 2016; Arumainayagam et al., 2021), relevant for different astrochemical environments, from the ISM to planetary atmospheres or soil. These interactions can initiate different processes within molecules, depending on the photon energies, and can lead to electronic excitation within neutral molecules or create ions by ejecting off the valence or even inner-layer electrons. Interactions with photons can lead to the creation of new molecules, or their damage, but are also relevant for extending observable spectral ranges (Öberg, 2016).

Extensive laboratory experimental investigation of the photoabsorption spectra of benzonitrile recorded using synchrotron radiation in $35^{\prime }000-90^{\prime }000cm^{-1}$ (4.3–11.1 eV, 0.111–0.286 μm ), which encompasses several neutral and ionic excited states, as recently reported by Rajasekhar et al. (2022). From a computational perspective, these processes can be simulated by means of vibronic computations (Bloino et al., 2016; Barone et al., 2021), which have been demonstrated to allow us to decipher a broad range of energies by the combination of two-state electronic transitions for a series of halogenated benzene (Palmer et al., 2015a; b). Moreover, first-principle spectral simulations also allow us to obtain reference data for unstable species difficult to study in the laboratories and to improve the resolution and predict spectra at a broad range of temperatures (Zhao et al., 2021). Computational spectroscopy studies combining anharmonic vibrational and vibronic simulations covering the broad range from MIR at approximately 20 μm up to even 20 nm in a high-energy photoelectron range can be extended toward other cyano-astroCOMs, supporting their possible detection.

The most relevant are those based on aromatic systems, such as 1- and 2-cyanocyclopentadiene (McCarthy et al., 2021; Lee et al., 2021), and 1- and 2-cyanonaphthalene (McGuire et al., 2021), which have been already discovered in TMC-1. However, it can be expected that similar accuracy can also be obtained for aliphatic systems, for which several conformers can be present (Barone et al., 2013; 2015b). This situation was highlighted by the recent discovery of five cyano-derivatives of propene ( ${CH}_{2}$ CHCH_3_), based on the QUIJOTE line survey of TMC-1 (Cernicharo et al., 2022). Such computations, including electronic spectra for benzonitrile and MIR to PES spectra for other C≡N -astroCOM species, are deferred to subsequent works, within the framework of development of the COSY-ASTRO dataset (COSY COST Action CA21101, 2024).

## Data Availability

The original contributions presented in the study are included in the article/Supplementary Material; further inquiries can be directed to the corresponding author.
