# Supplementary material for: Toward the identification of cyano-astroCOMs via vibrational features: benzonitrile as a test case
Source: Front Chem. 2024 Sep 3;12:1439194. doi: 10.3389/fchem.2024.1439194 (PMC11408737; doi:10.3389/fchem.2024.1439194)
Supplement: Supplementary file 1 [file Table1.docx]

Table S1. Structural parameter of benzonitrile (bond lengths in Å, angles in deg)

|  | r_e_^SE^ *^a^* | r_e_^BO^ *^b^* | CCSD(T) *^c^* | rDSD | rDSD | B3LYPD | B3LYPD |
| --- | --- | --- | --- | --- | --- | --- | --- |
|  |  |  | ANO1 | SNSD | junTZ | SNSD | junTZ |
| **Bond Lengths** | | | | | | | |
| C1-C2 | 1.3968 | 1.3962 | 1.40117 | 1.4039 | 1.3993 | 1.4025 | 1.3981 |
| C2-C3 | 1.3884 | 1.3882 | 1.39336 | 1.3954 | 1.3905 | 1.3912 | 1.3862 |
| C3-C4 | 1.3917 | 1.3917 | 1.39668 | 1.3989 | 1.3941 | 1.3956 | 1.3907 |
| C4-C6 | 1.3917 | 1.3917 | 1.39668 | 1.3989 | 1.3941 | 1.3956 | 1.3907 |
| C6-C5 | 1.3884 | 1.3882 | 1.39336 | 1.3954 | 1.3905 | 1.3912 | 1.3862 |
| C5-C1 | 1.3968 | 1.3962 | 1.40117 | 1.4039 | 1.3993 | 1.4025 | 1.3981 |
| C1-C12 | 1.4347 | 1.4359 | 1.43933 | 1.4401 | 1.4360 | 1.4327 | 1.4287 |
| C2-H8 | 1.0780 | 1.0803 | 1.08226 | 1.0875 | 1.0826 | 1.0846 | 1.0801 |
| C3-H7 | 1.0799 | 1.0803 | 1.08243 | 1.0878 | 1.0830 | 1.0853 | 1.0808 |
| C4-H9 | 1.0800 | 1.0806 | 1.08275 | 1.0881 | 1.0832 | 1.0856 | 1.0811 |
| C6-H11 | 1.0799 | 1.0803 | 1.08243 | 1.0878 | 1.0830 | 1.0853 | 1.0808 |
| C5-H10 | 1.0780 | 1.0803 | 1.08226 | 1.0875 | 1.0826 | 1.0846 | 1.0801 |
| C12-N13 | 1.1582 | 1.1583 | 1.16458 | 1.1708 | 1.1629 | 1.1608 | 1.1523 |
| \|MAX\| | - | 0.0023 | 0.0064 | 0.0126 | 0.0047 | 0.0066 | 0.0059 |
| MAE | - | 0.0007 | 0.0043 | 0.0080 | 0.0030 | 0.0045 | 0.0022 |
| **Angles** | | | | | | | |
| C1C2C3 | 119.42 | 119.52 | 119.54 | 119.58 | 119.55 | 119.69 | 119.67 |
| C2C3C4 | 120.27 | 120.13 | 120.13 | 120.16 | 120.17 | 120.18 | 120.18 |
| C3C4C6 | 120.07 | 120.22 | 120.19 | 120.14 | 120.16 | 120.13 | 120.16 |
| C4C6C5 | 120.27 | 120.13 | 120.13 | 120.16 | 120.17 | 120.18 | 120.18 |
| C1C5C6 | 119.42 | 119.52 | 119.54 | 119.58 | 119.55 | 119.69 | 119.67 |
| C2C1C5 | 120.55 | 120.50 | 120.46 | 120.37 | 120.41 | 120.13 | 120.13 |
| C1C2H8 | 119.77 | 119.61 | 119.61 | 119.60 | 119.64 | 119.54 | 119.57 |
| C3C2H8 | 120.81 | 120.87 | 120.85 | 120.82 | 120.81 | 120.77 | 120.76 |
| C2C3H7 | 119.63 | 119.72 | 119.71 | 119.69 | 119.70 | 119.68 | 119.69 |
| C4C3H7 | 120.10 | 120.16 | 120.16 | 120.14 | 120.14 | 120.15 | 120.13 |
| C3C4H9 | 119.96 | 119.89 | 119.90 | 119.93 | 119.92 | 119.93 | 119.92 |
| C6C4H9 | 119.96 | 119.89 | 119.90 | 119.93 | 119.92 | 119.93 | 119.92 |
| C4C6H11 | 120.10 | 120.16 | 120.16 | 120.14 | 120.14 | 120.15 | 120.13 |
| C5C6H11 | 119.63 | 119.72 | 119.71 | 119.69 | 119.70 | 119.68 | 119.69 |
| C6C5H10 | 120.81 | 120.87 | 120.85 | 120.82 | 120.81 | 120.77 | 120.76 |
| C1C5H10 | 119.77 | 119.61 | 119.61 | 119.60 | 119.64 | 119.54 | 119.57 |
| C5C1C12 | 119.72 | 119.75 | 119.77 | 119.81 | 119.80 | 119.93 | 119.93 |
| C2C1C12 | 119.72 | 119.75 | 119.77 | 119.81 | 119.80 | 119.93 | 119.93 |
| \|MAX\| | - | 0.16 | 0.16 | 0.18 | 0.15 | 0.42 | 0.42 |
| MAE | - | 0.09 | 0.09 | 0.09 | 0.08 | 0.13 | 0.13 |

^a^ Semi-experimental r_e_^SE^ from Ref (Rudolph et al., 2013), obtained by combining the experimental ground-state rotational constants for set of isotopologues with rovibrational corrections derived from cubic force fields determined by B3LYP level.

^b^ Theoretical best estimated r_e_^BO^ from Ref (Rudolph et al., 2013), obtained by means of a composite ab initio approach based on CCSD(T) and MP2 all-electron optimizations with basis sets up to quintuple-zeta quality.

^c^ Theoretical structure obtained at the CCSD(T)/ANO-1 level from Ref (Zdanovskaia et al., 2022).

^d^ Largest absolute (|MAX|) and average abolute errors (MAE) of the bond length and bond angles compared to the r_e_^SE^ from Ref (Rudolph et al., 2013).

S2: Geometry of the benzonitrile, optimized in GAUSSIAN 16 at rDSD/junTZ level of theory.

| C | 0.0000 | 0.0000 | 0.6048 |
| --- | --- | --- | --- |
| C | 0.0000 | 1.2143 | -0.0905 |
| C | 0.0000 | 1.2083 | -1.4810 |
| C | 0.0000 | 0.0000 | -2.1763 |
| C | 0.0000 | -1.2143 | -0.0905 |
| C | 0.0000 | -1.2083 | -1.4810 |
| H | 0.0000 | 2.1467 | -2.0216 |
| H | 0.0000 | 2.1465 | 0.4599 |
| H | 0.0000 | 0.0000 | -3.2596 |
| H | 0.0000 | -2.1465 | 0.4599 |
| H | 0.0000 | -2.1467 | -2.0216 |
| C | 0.0000 | 0.0000 | 2.0408 |
| N | 0.0000 | 0.0000 | 3.2037 |

S3: IR spectrum computed in GAUSSIAN 16 at GVPT2//revDSD/junTZ//B3LYP/SNSD level.

Attached file: c6h5cn_GVPT2_peak_info.csv
